# In-office Bone-Anchored Hearing Implants via Minimally Invasive Punch Technique in a Veteran Population

**DOI:** 10.1177/01945998221086841

**Published:** 2022-03-29

**Authors:** Jackson King, Isabella Leon, Lane Squires

**Affiliations:** 1 Department of Otolaryngology–Head and Neck Surgery, University of California–Davis Medical Center, Sacramento, California, USA; 2 Veterans Affairs Northern California Healthcare System, Sacramento, California, USA

**Keywords:** bone-anchored hearing implant, minimally invasive, otology, Veteran Affairs

## Abstract

**Objective:**

Describe the feasibility and safety of completing bone-anchored hearing implants via the minimally invasive punch technique in the in-office setting.

**Study Design:**

This single-institution case series included 20 patients who underwent in-office bone-anchored hearing implant placement under local anesthesia from 2018 to 2021.

**Setting:**

Veterans Affairs Northern California Healthcare System.

**Methods:**

Following completion of the case series, patients were retrospectively surveyed regarding their satisfaction with this approach via a modified SSQ-8 (Surgical Satisfaction Questionnaire) to fit our purposes.

**Results:**

A total of 23 implants were completed in the in-office setting on 20 patients. Intra- and postoperative complication rates, including skin changes, irritation, infection, and poor wound healing, were similar to or better than currently published complication rates in the literature. In addition, patients reported overwhelmingly positive responses on the SSQ-8, almost universally stating that they were “very satisfied” with their clinic experience.

**Conclusion:**

This case series suggests that it is feasible and safe to complete this procedure in the clinic under local anesthesia, but further prospective studies are needed to evaluate this in a more generalized population.

Since its introduction in the 1970s, bone-anchored hearing implants (BAHIs) have rapidly developed into a viable alternative treatment option for patients with various forms of conductive, mixed, or single-sided hearing loss.^1,2^ They primarily consist of an osseointegrated titanium screw embedded into the mastoid or squamous portion of the temporal bone and connected to a cutaneous abutment through which a sound processor is attached. Sound is then transmitted via bone conduction from the temporal bone to the inner ear or, in the case of unilateral deafness, to the contralateral intact cochlea.^3^

The traditional surgical technique for implantation of a BAHI involves a linear or U-shaped incision placed in the retroauricular scalp approximately 5 to 7 cm from the external auditory canal. It was originally hypothesized that heavy soft tissue undermining and debulking could be used to obtain a hairless immobile implant site that is conducive for a tight bone-implant interface.^4^ However, this resulted in complications such as periabutment inflammation, skin reactions, and soft tissue overgrowth, which led to increased interest in an improved surgical approach.^5-7^ A more modern approach advocated for less soft tissue undermining, resulting in reduced adverse skin reactions, operative length, and improved cosmesis.^8-10^

In 2015, Oticon Medical introduced its Food and Drug Administration–approved minimally invasive Ponto surgery (MIPS), which utilizes a specially designed surgical kit and results in a scarless implant via a punch technique.^11^ In comparison with the retroauricular linear incision without tissue reduction, MIPS has been demonstrated to decrease surgical time with an associated decrease in cost >$400. Patients undergoing the MIPS technique have also experienced an improved cosmetic appearance and decreased loss of skin sensation.^12-14^

The minimally invasive punch technique has traditionally been completed in the operating room (OR) with sedation, yet we were interested in utilizing the minimally invasive BAHI option in our office procedure room when possible to reduce the ever-present OR burden, as well as to limit unnecessary anesthesia risks for our veteran population. Shifting these procedures to this setting has proved tremendously beneficial.^15^ Herein, we aim to review the feasibility, safety, and patient experience when performing in-office minimally invasive implants under local anesthesia in a veteran population.

## Description of Procedure in Office Setting

All surgical procedures were performed with the MIPS technique, with procedure specifications made for utilization of Oticon medical implants. In brief, the implant site is identified approximately 5.5 to 6.0 cm posterior and superior to the external acoustic meatus. Scalp thickness is measured at the desired implant location with a hypodermic needle, which is immediately used to inject 1 to 2 mL of 1% lidocaine with 1:100,000 epinephrine. The injection occurs first at the temporal bone and then is slowly withdrawn to ensure that the periosteum and subcutaneous tissues are all addressed at the site. A 5-mm-diameter punch biopsy is then used to make a circular incision up to the bone surface. Through the circular incision, the scalp tissue, including the periosteum, is stripped and removed. The drill cannula is seated perpendicular to the bone, and a 4-mm-deep recipient site for the implant is created. A countersink drill bit may be employed as needed to allow for appropriate fitting of the specific implant size. Continuous irrigation is used during the drilling process to minimize heat of the surrounding bone and assist with removal of debris. The cannula is removed once the implant is placed, and a healing cap and dressing are placed until removal at the surgical follow-up visit.^16^

## Materials and Methods

This study was approved by the Veterans Affairs (VA) Institutional Review Board (1608453). From 2018 to 2021 at the VA Northern California Healthcare System, the study author (LS) completed 23 BAHI implants on 20 patients utilizing the MIPS approach in the office, under local anesthesia without sedation. One patient received bilateral implants, and there were 2 revision cases. The 2 revision cases were done in the OR for factors unrelated to patient tolerance, and these were excluded in our in-office complication numbers. Rates of intra- and postoperative complications for our office procedures were compared with the published literature. In addition, the time associated with minimally invasive implantation for both the patient and the provider was recorded and compared between the in-office (n = 23) and OR (n = 2) cases.

Retrospectively, patients were surveyed regarding their experience with the procedure via an adapted version of the Surgical Satisfaction Questionnaire (SSQ-8; Appendix 1, available online). The original SSQ-8 is a validated tool for assessment of postprocedural patient satisfaction initially described in the urogynecologic literature.^17^

## Results

Of the 23 implants placed in the office setting under local anesthesia, 8 were completed with an abutment length of 9 mm, 9 with 12 mm, and 6 with 14 mm. All 20 patients were male with an average age of 69.6 years; 5 (25%) were active smokers; and 13 (65%) had undergone prior ear surgery. Fifteen implants were completed on the left side and 8 on the right, with a mean postprocedure follow-up of 11 months.

Intraoperatively, there were no incidents of dura mater exposure or cerebrospinal fluid leak (**Table 1**). There was 1 case of active bleeding from the bone that resolved with placement of the osseointegrated screw. Postoperatively, 2 of 20 patients (10%) experienced skin reactivity at the abutment site with small areas of erythema and granulation tissue that resolved without further treatment. There were no episodes of moderate edema, erythema, or gross infection. The screw/abutment became dislodged in 4 of the 23 in-office cases (17.4%) in 3 patients. One patient underwent 2 revisions secondary to repeated trauma to the area, resulting in multiple dislodgements. One patient underwent hardware removal secondary to dissatisfaction with hearing after the procedure and returned to hearing aid use. An additional patient has alternated between use of his original air conduction hearing aid and the bone conduction device.

**Table 1.** Periprocedural Complication Rates in Present Study vs Published Literature.^a^

	VA NCHCS	Literature^12-14,22^
Intraoperative events		
Dura mater exposure	0	3.9
Cerebrospinal fluid leak	0	1.3
Active bleeding from the bone	4	14.3
Postoperative complications		
Small areas of granulation tissue/minor irritation	8.7	12
Moderate edema, erythema, or gross infection	0	4.4
Screw/abutment dislodgement	16.6	12

Abbreviation: VA NCHCS, Veterans Affairs Northern California Healthcare System.

a Values are presented as percentages.

Of the 20 patients who initially received an implant and were contacted for SSQ-8 completion, 4 were deceased by the time of survey collection. Of the remaining 16 patients, 14 completed the survey. The SSQ-8 was organized such that patients could respond to questions in a Likert scale format ranging from *very satisfied* to *unsatisfied*. Responses indicated almost universal *very satisfied* for all 6 domains of the survey questionnaire, with the exception of 1 patient reporting *neutral* for the question of satisfaction with pain at home postoperatively (Appendix 1, available online). Four patients reported *not applicable* for the question of satisfaction with time to return to work. All 14 patients indicated that they would be willing to undergo the procedure again and would recommend it to someone else.

**Figure 1.**
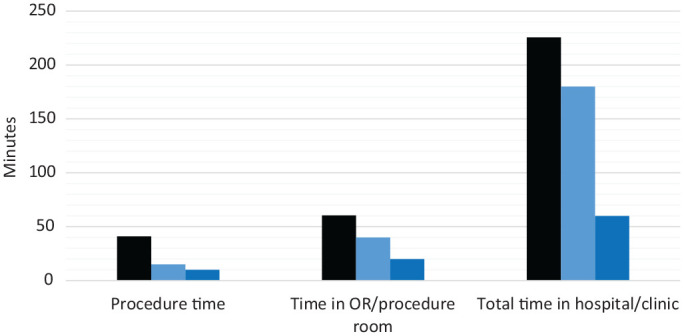
Time associated with minimally invasive implantation for both patient and provider. Black, traditional surgical technique completed in the OR. Light blue, MIPS completed in the OR. Dark blue, MIPS completed in the clinic. MIPS, minimally invasive Ponto surgery; OR, operating room.

The time associated with minimally invasive implantation for the patient and the provider is noted in **Figure 1**. This includes the time needed for the OR vs the clinic for BAHI procedures at our institution. Additionally, our time data were compared with available published times for the traditional technique of implantation via a retroauricular incision.^15^ When minimally invasive punch BAHI procedures were compared, there was a 5-minute reduction in procedural time in the clinic vs the OR. Furthermore, there was a 20-minute reduction of time in the OR vs the office and a 120-minute reduction in time spent in the hospital vs the clinic.

## Discussion

With the aim of maintaining quality assurance in the setting of reduced time and facility charges, there has been a growing body of literature within otolaryngology describing the movement of surgical procedures from the OR to the office setting, with favorable results in safety outcomes and patient satisfaction.^18,19^

To date, no published data exist examining the feasibility, safety, and time savings for implantation of BAHI in an office setting. In our case series, we observed a substantial decrease in the total time spent by the patient and physician when comparing BAHI via a minimally invasive punch procedure in the OR vs the clinic. This time reduction was also likely associated with a cost reduction, given the elimination of the need for an anesthesia provider and associated OR staff, as well as other hospital-based resources. We found it challenging, however, to derive any specific data on our cost savings utilizing this technique in an office setting vs the more traditional OR setting, given the inherent limitations of the VA system regarding procedural billing. Sardiwalla et al^20^ performed a cost comparison of a minimally invasive punch technique with traditional approaches for percutaneous BAHI placement. They demonstrated a cost reduction of $456.83 with a time reduction of 61 minutes when comparing a minimally invasive punch technique with an open approach. Given that the time needed to perform a punch implant procedure in the clinic is nearly half of what is required in the OR, a surgeon could theoretically perform twice as many BAHI procedures in 1 day and/or could increase one’s availability to see other patients or pursue other endeavors. The patients additionally benefit from the time reduction permitted by in-office BAHI implantations. Patients can forgo the additional wait times and effort required for pre- and postoperative evaluations prior to an OR-based procedure, potentially avoiding extra time away from work or other personal activities.

Our patients’ satisfaction with this option can be inferred from the overwhelmingly positive responses to the modified SSQ-8, indicating “yes” on the question regarding if they would have the procedure done in the same way again. The patient’s qualitative perspectives from this study were similar to the responses found by Sardiwalla et al,^21^ whose Canadian group used a modified SSQ-8 to assess quality assurance in patients undergoing in-office BAHA procedures with the MIPS technique. In their analysis of the modified SSQ-8, the Likert scale–based results ranged from 1 to 5 (1 = satisfied, 3 = neutral, 5 = unsatisfied) in all categories, which evaluated result of surgery, recovery, abutment length satisfaction, overall satisfaction, and recommendation of MIPS to others who would benefit. These were remarkably similar to the positive responses received from our patient population of veterans (Appendix 1, available online).^21^

Adverse outcomes associated with implantation were also similar to currently published risks demonstrating the feasibility and safety of this approach. Intraoperatively, our complication rates were similar to or better than rates described in the literature (**Table 1**).^22^

Skin reactions are a well-described, foreseen occurrence in BAHIs but are not considered a complication.^23^ Mild skin reactions were seen in only 2 patients, neither of whom required revision. The absence of major complications of soft tissue reaction may be secondary to the choice of using the minimally invasive punch procedure, as there is less soft tissue mobilization in this surgical technique vs the open approach with a linear incision.^24^ Our study also had 4 cases of screw and abutment dislodgement, all of which were associated with blunt trauma directly to the abutment in the immediate postprocedure period (<1 month). This trauma consisted repetitive direct injury from an electric haircutting razor, a basketball taken directly to the post twice in a referee, and a swinging tree branch. None of these dislodgements were suspected to be caused by a surgical error or the fact that the procedure was performed in an office setting rather than the OR.

Based on the analysis of complications, time reduction, and patient satisfaction, our study demonstrates that implantation of BAHIs via the punch technique can be performed safely and effectively in the office setting and should be considered an option for patients. It should be noted that appropriate patient selection for the minimally invasive BAHI option in the office is paramount. One must consider the comorbidities of the patients when selecting candidates for in-office procedures, as well as the their ability to tolerate an awake procedure under local anesthetic only.

Several limitations to this study warrant discussion. This was a single-institution case series that involved exclusively a veteran population, which in our case series was 100% male. We were unable to compare our data points with those of a veteran population undergoing this procedure in the OR setting, given the success rate and ease of completing the procedure in the office; thus, further research is needed to investigate how patient satisfaction and postoperative pain may compare between the groups, especially considering the differences in anesthesia. Also, while many validated patient satisfaction questionnaires exist, the heterogeneity of their construct and application may make it difficult to compare across studies. As this was a retrospective study, the time interval from the procedure to completion of the satisfaction questionnaire varied significantly from patient to patient, which could introduce recall bias. Additional advantages or disadvantages may be borne out with a larger cohort or with longer follow-up times. However, given the absence of major complications, coupled with the mean follow-up of 11 months and the overwhelmingly positive responses from the modified SSQ-8, it is unlikely that longer follow-up will yield substantially different findings. While there is direct applicability of this study to other veteran populations, additional work is needed to generalize the results of this study to a more widespread population.

## Conclusion

Our case series suggests that BAHI placement via the minimally invasive punch technique can feasibly be completed in the in-office setting with similar complication rates to the OR, saving considerable time and resources for the health system and the patient. Further studies will be needed to evaluate this in a more widespread population outside the VA health system.

## Supplemental Material

sj-docx-1-oto-10.1177_01945998221086841 – Supplemental material for In-office Bone-Anchored Hearing Implants via Minimally Invasive Punch Technique in a Veteran Population Click here for additional data file. Supplemental material, sj-docx-1-oto-10.1177_01945998221086841 for In-office Bone-Anchored Hearing Implants via Minimally Invasive Punch Technique in a Veteran Population by Jackson King, Isabella Leon and Lane Squires in Otolaryngology–Head and Neck Surgery
