# Supplementary figures and images for: In-office Bone-Anchored Hearing Implants via Minimally Invasive Punch Technique in a Veteran Population
Source: Otolaryngol Head Neck Surg. 2022 Mar 29;167(6):959–63. doi: 10.1177/01945998221086841 (PMC9720705; doi:10.1177/01945998221086841)

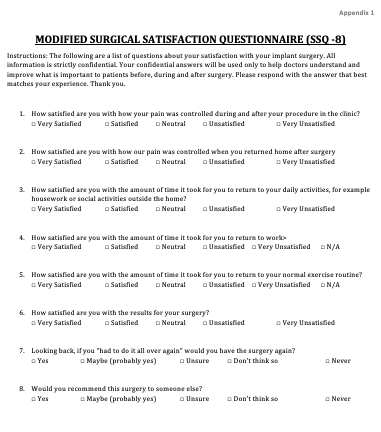

Supplement: sj-docx-1-oto-10.1177_01945998221086841 – Supplemental material for In-office Bone-Anchored Hearing Implants via Minimally Invasive Punch Technique in a Veteran Population [file sj-docx-1-oto-10.1177_01945998221086841.docx]
